# Supplementary figures and images for: Changes in social environment due to the state of emergency and Go To campaign during the COVID-19 pandemic in Japan: An ecological study
Source: PLoS One. 2022 Apr 27;17(4):e0267395. doi: 10.1371/journal.pone.0267395 (PMC9045837; doi:10.1371/journal.pone.0267395)

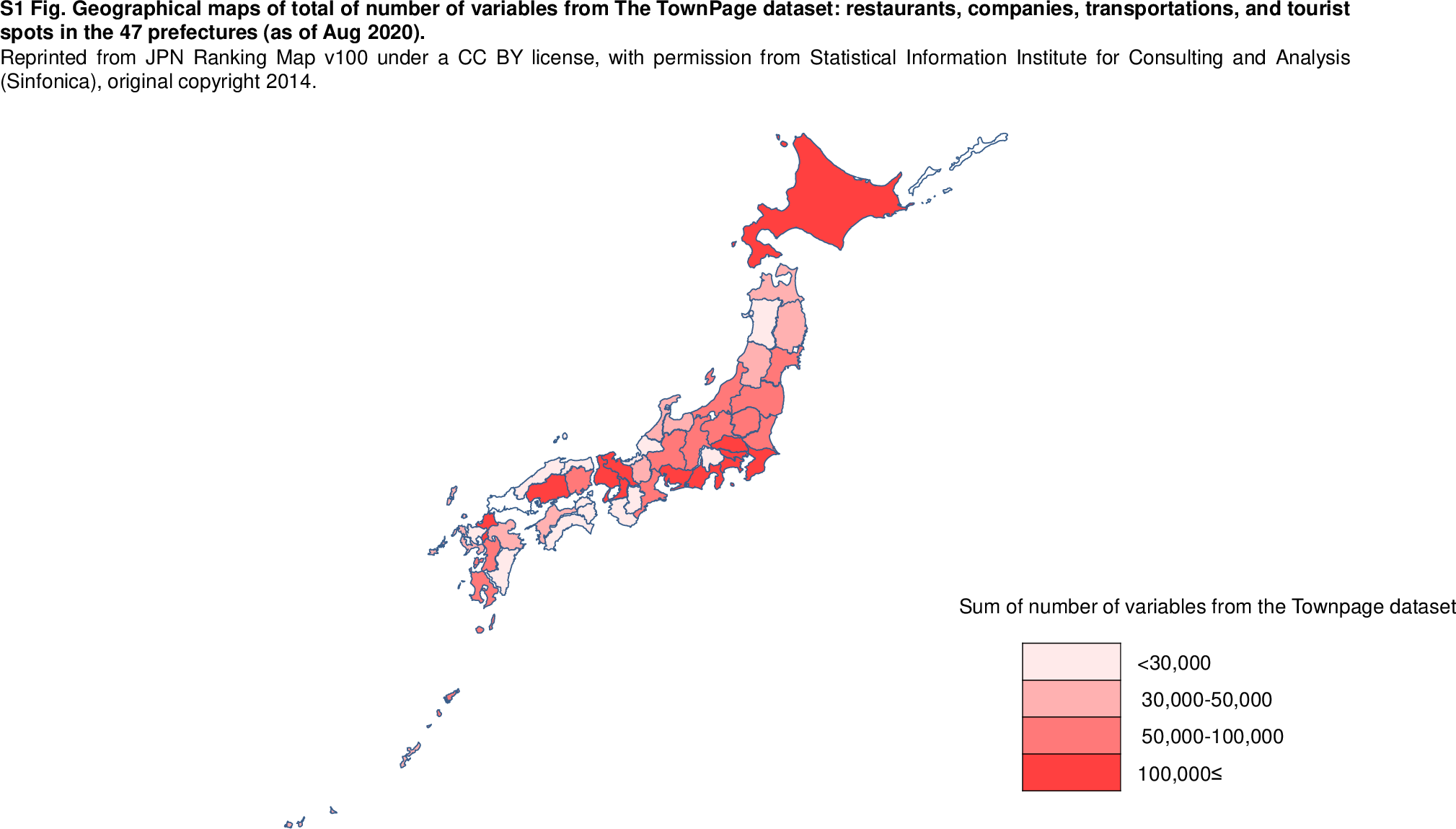

Supplement: S1 Fig — (TIF) [file pone.0267395.s001.tif]

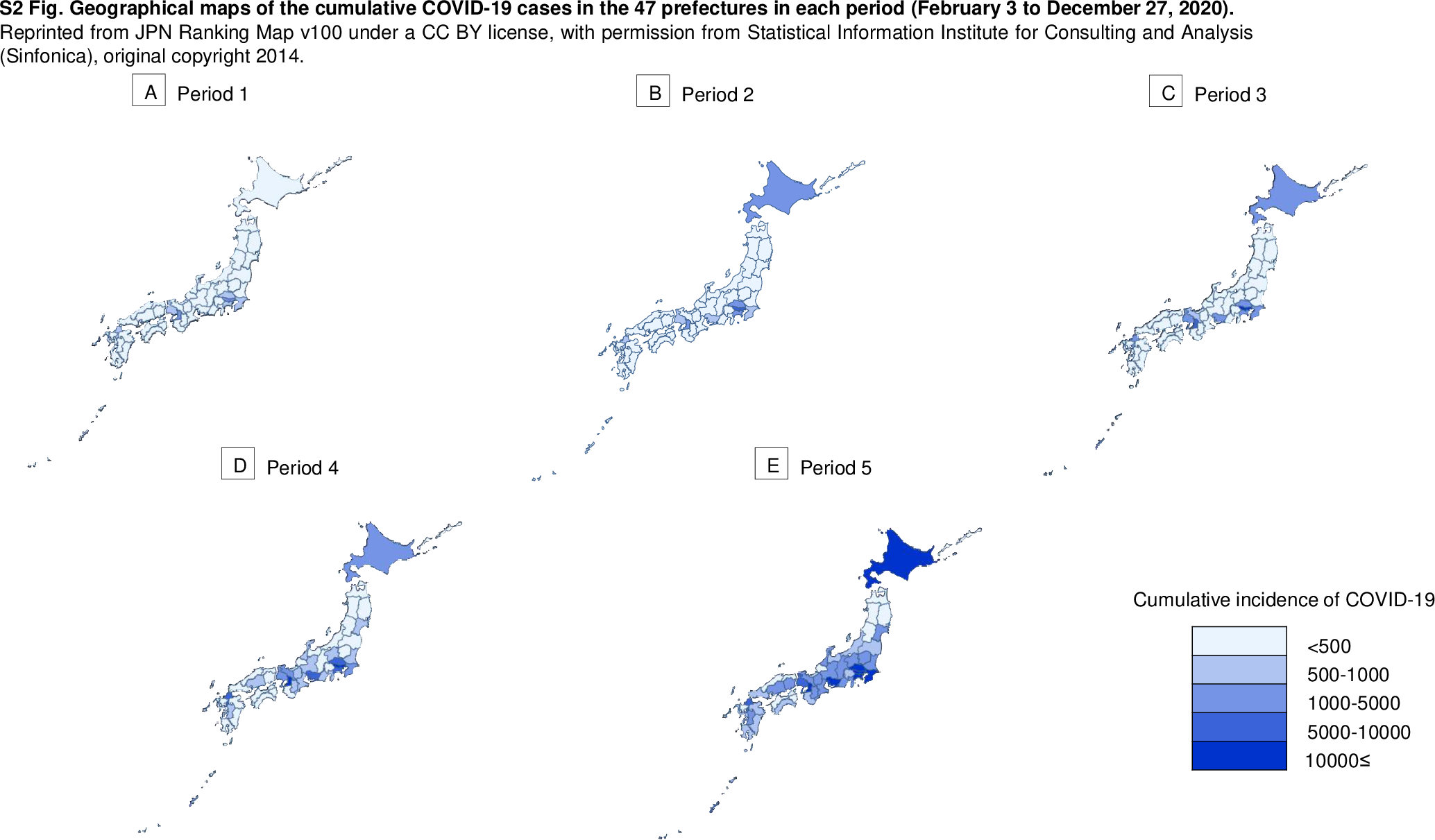

Supplement: S2 Fig — (TIF) [file pone.0267395.s002.tif]

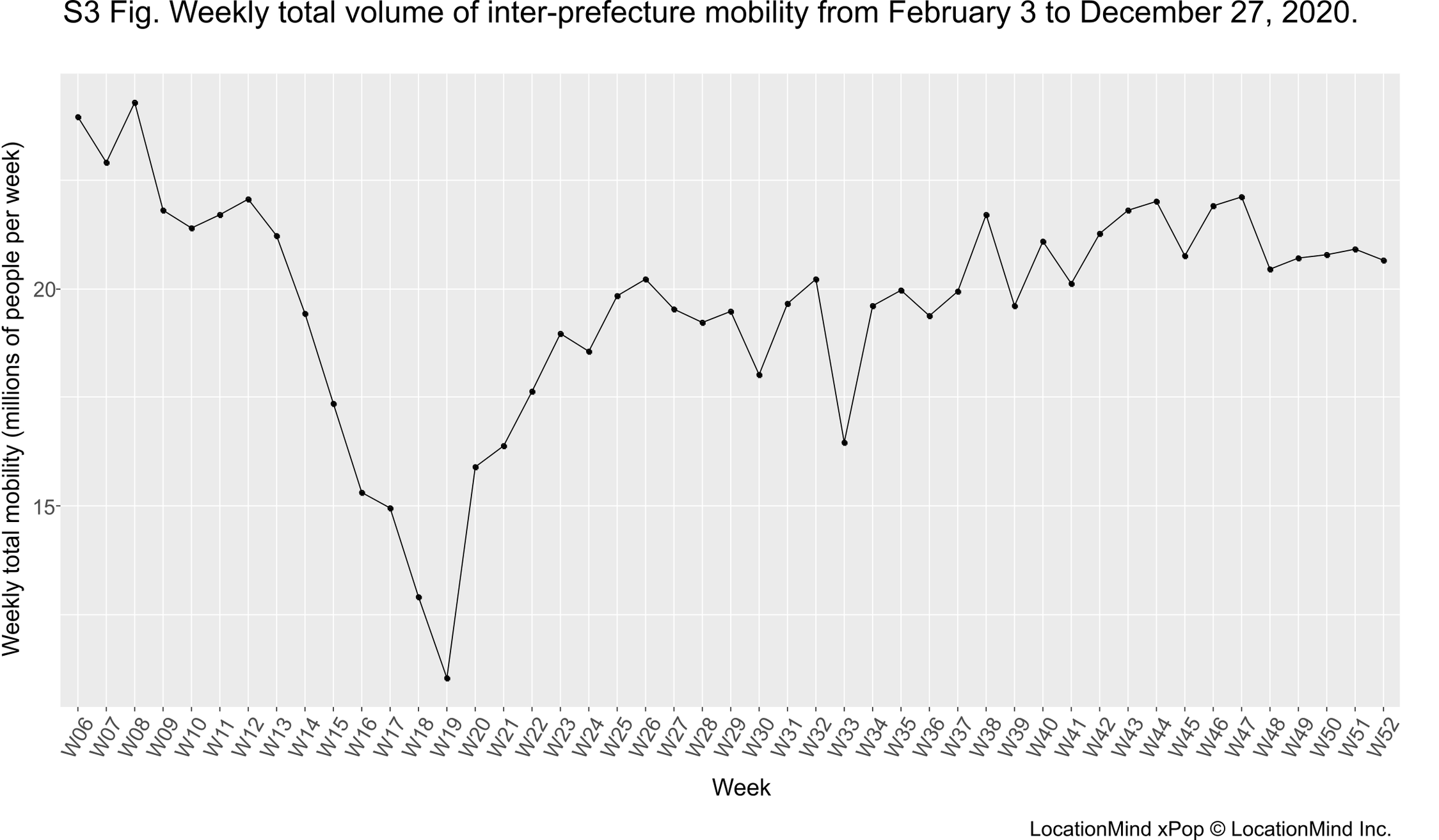

Supplement: S3 Fig — (TIF) [file pone.0267395.s003.tif]

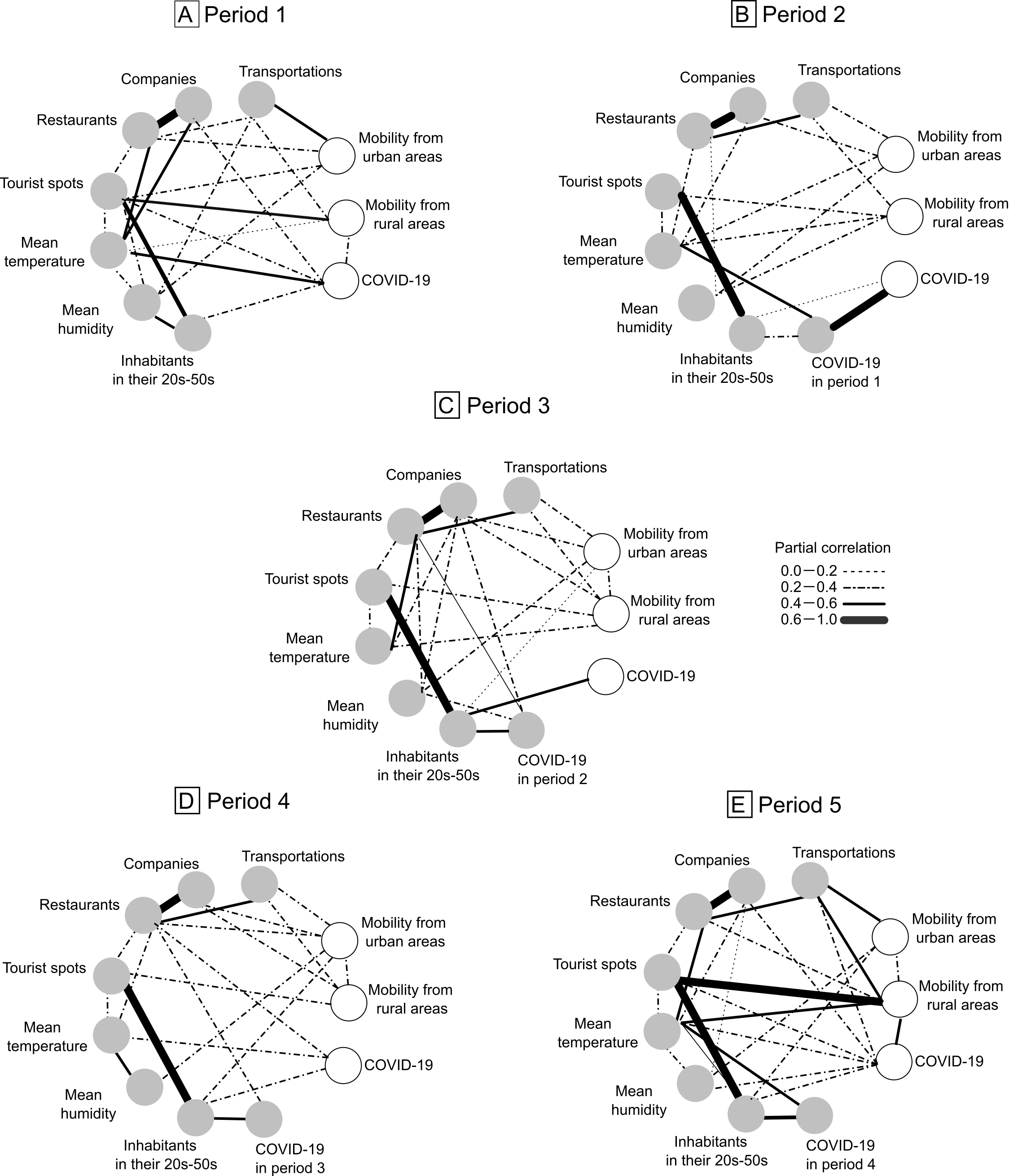

Supplement: S4 Fig — (TIF) [file pone.0267395.s004.tif]

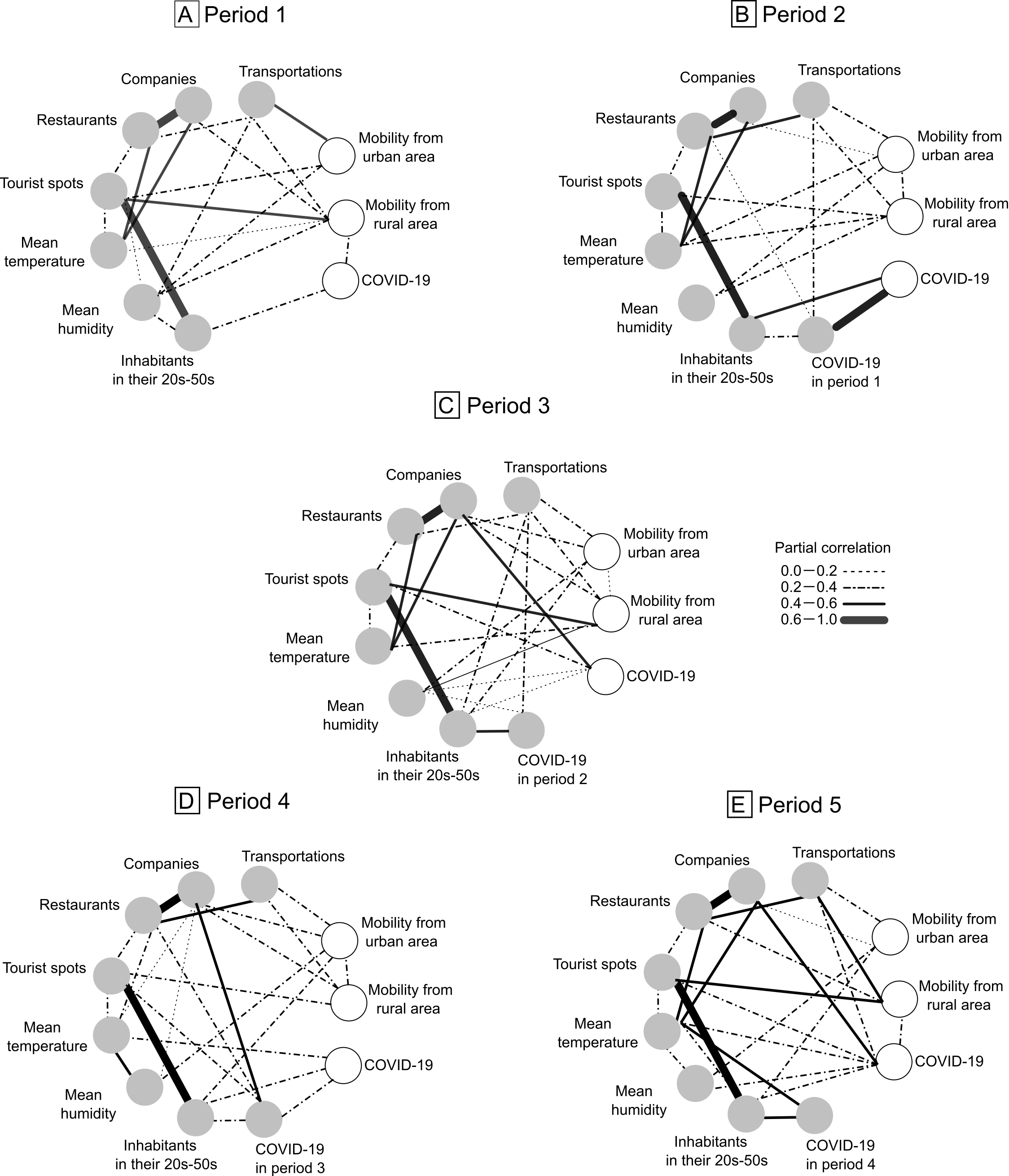

Supplement: S5 Fig — (TIF) [file pone.0267395.s005.tif]
