# Supplementary material for: Changes in social environment due to the state of emergency and Go To campaign during the COVID-19 pandemic in Japan: An ecological study
Source: PLoS One. 2022 Apr 27;17(4):e0267395. doi: 10.1371/journal.pone.0267395 (PMC9045837; doi:10.1371/journal.pone.0267395)
Supplement: S1 Table — (DOCX) [file pone.0267395.s006.docx]

**S1 Table. Initial nationwide public health measures during the COVID-19 pandemic in Japan and other countries.**

| **Country** | **Initial government measure in 2020** | **Restrictions on social distancing and mobility to prevent the COVID-19 pandemic** |
| --- | --- | --- |
| **Japan[1, 2]** | State of emergency | Request to avoid unnecessary outings (self-restriction)  Request to close restaurants and bars that serve alcohol and to shorten business hours  Recommendations on school closure, workplace closure, and cancellations of public events  No compulsory restrictions on public transportation and domestic travel |
| **China[1, 3]** | Wuhan Lockdown | Lockdown of limited areas with COVID-19 infections  Reliance on local governments’ decisions regarding lockdown implementation  Closure of schools, workplaces, and public transportation  Restrictions on domestic and international travel |
| **United States[3]** | Stay-at-home orders | Reliance on state-level regulations  Closure of schools, workplaces, non-essential businesses, and public transportation  Restrictions on out-of-state travel |
| **Italy[3]** | Lockdown | Nationwide lockdowns  Outdoor activities banned  Closure of nonessential businesses  Restrictions on public transportation |

**References:**

1. Yan B, Zhang X, Wu L, Zhu H, Chen B. Why Do Countries Respond Differently to COVID-19? A Comparative Study of Sweden, China, France, and Japan. The American Review of Public Administration. 2020;50(6-7):762-9. doi:10.1177/0275074020942445.

2. Prime Minister's Office of Japan. COVID-19 Control Headquarters (27th meeting). [cited 2020 October 1]. Available from: https://www.kantei.go.jp/jp/98_abe/actions/202004/07corona.html.

3. Ren X. Pandemic and lockdown: a territorial approach to COVID-19 in China, Italy and the United States. Eurasian Geography and Economics. 2020;61(4-5):423-34. doi:10.1080/15387216.2020.1762103.
