# Supplementary material for: Changes in social environment due to the state of emergency and Go To campaign during the COVID-19 pandemic in Japan: An ecological study
Source: PLoS One. 2022 Apr 27;17(4):e0267395. doi: 10.1371/journal.pone.0267395 (PMC9045837; doi:10.1371/journal.pone.0267395)
Supplement: S2 Table — (PDF) [file pone.0267395.s007.pdf]

S2 Table. Process of recursive covariance selection in graphical modeling of period 1 in main analysis.

A. Initial status of model selection

Deviance = N/A, Degrees of freedom = N/A, GFI = 1.00

Group of factors | Group of outcomes

	Restaurant	Transportations	Companies	Tourist spots	Inhabitants in ther 20s-50s	Mean temperature	Mean humidity	COVID-19	Mobility from urban areas	Mobility fom rural areas
Restaurant										
Transportations	0.35294									
Companies	0.71713	0.05441								
Tourist spots	0.36210	-0.07550	-0.09015							
Inhabitants in ther 20s-50s	0.22280	-0.14991	-0.22554	-0.69994						
Mean temperature	0.56986	-0.00751	-0.55257	-0.29677	-0.14305					
Mean humidity	0.02807	0.2543	-0.13325	-0.12312	-0.36547	-0.01678				
COVID-19	0.27221	-0.06689	-0.13984	-0.08878	0.34761	-0.13220	-0.12512			
Mobility from urban areas	-0.03937	-0.34772	-0.12029	-0.16278	0.10869	0.11973	-0.24134	-0.01556		
Mobility from rural areas	0.09669	-0.28362	0.21313	-0.51375	-0.13584	-0.25596	-0.30450	-0.10978	0.15971	

Lower triangular matrix shows partial correlation coefficient.
Upper triangular matrix is ommited, as it is symmetrical to lower triangular matrix.

B. Interim status of model selection

Deviance = 4.478 , Degrees of freedom = 16 N/A, GFI = 0.99

Group of factors | Group of outcomes

	Restaurant	Transportations	Companies	Tourist spots	Inhabitants in ther 20s-50s	Mean temperature	Mean humidity	COVID-19	Mobility from urban areas	Moblity from rural areas
Restaurant							-0.04296		0.01298	0.01591
Transportations	0.36385		0.01058	-0.02398		-0.00415		-0.02540		
Companies	0.71914	0.00001		-0.01768			-0.08638			
Tourist spots	0.28139	-0.00001	0.00000					-0.00490		
Inhabitants in ther 20s-50s	0.16290	-0.12974	-0.13764	-0.69888		-0.01600			0.02407	
Mean temperature	0.55434	0.00000	-0.54063	-0.25384	-0.10718		0.02822		0.05038	
Mean humidity	-0.00001	0.21472	0.00000	-0.17605	-0.36435	0.00000		-0.06482		
COVID-19	0.24107	-0.00010	-0.14644	0.00000	0.40310	-0.10312	-0.00001		0.02750	-0.01456
Mobility from urban areas	0.00000	-0.35632	-0.15774	-0.25375	0.00001	0.00000	-0.28303	0.00000		
Mobility from rural areas	0.00000	-0.27661	0.26912	-0.48463	-0.14381	-0.19336	-0.29710	0.00001	0.11727	

Lower triangular matrix shows partial correlation coefficient.
Upper triangular matrix is ommited, as it is symmetrical to lower triangular matrix.

C. Final status of model selection

Deviance = 16.79 , Degrees of freedom = 26 N/A, GFI = 0.96

Group of factors | Group of outcomes

	Restaurant	Transportations	Companies	Tourist spots	Inhabitants in ther 20s-50s	Mean temperature	Mean humidity	COVID-19	Mobility from urban areas	Moblity from rural areas
Restaurant										
Transportations	0.37936									
Companies	0.72989	0.00000								
Tourist spots	0.25935	0.00001	0.00000							
Inhabitants in ther 20s-50s	0.00000	-0.00001	-0.00001	-0.70467						
Mean temperature	0.56665	0.00000	-0.56313	-0.22796	0.00000					
Mean humidity	-0.00001	0.25006	0.00000	-0.18879	-0.39145	0.00000				
COVID-19	0.00000	0.00000	0.00001	0.00001	0.33644	0.00001	0.00001			
Mobility from urban areas	0.00000	-0.43997	-0.00001	-0.35265	0.00000	0.00000	-0.29057	0.00000		
Mobility from rural areas	0.00000	-0.29560	0.26508	-0.43342	0.00000	-0.19072	-0.27918	0.00000	0.00000	

Lower triangular matrix shows partial correlation coefficient.
Upper triangular matrix is ommited, as it is symmetrical to lower triangular matrix.
