# Supplementary material for: Changes in social environment due to the state of emergency and Go To campaign during the COVID-19 pandemic in Japan: An ecological study
Source: PLoS One. 2022 Apr 27;17(4):e0267395. doi: 10.1371/journal.pone.0267395 (PMC9045837; doi:10.1371/journal.pone.0267395)
Supplement: S3 Table — (DOCX) [file pone.0267395.s008.docx]

**S3 Table. The result of statistics in model selection in sensitivity analysis.**

|  | **0-day lag** | | | | | **7-day lag** | | | | | |
| --- | --- | --- | --- | --- | --- | --- | --- | --- | --- | --- | --- |
| **Period** | **1** | **2** | **3** | **4** | **5** | **1** | **2** | **3** | **4** | **5** |  |
| **Deviance** | 15.8 | 19.5 | 22.1 | 23.4 | 19.6 | 19.2 | 23.7 | 21.6 | 23.8 | 20.2 |  |
| **Degrees of freedom** | 22 | 29 | 31 | 33 | 272 | 26 | 32 | 29 | 31 | 28 |  |
| **GFI** ^a^ | 0.97 | 0.97 | 0.95 | 0.98 | 0.97 | 0.95 | 0.96 | 0.97 | 0.97 | 0.98 |  |

^a^ Goodness of Fit Index
