# Supplementary material for: Changes in social environment due to the state of emergency and Go To campaign during the COVID-19 pandemic in Japan: An ecological study
Source: PLoS One. 2022 Apr 27;17(4):e0267395. doi: 10.1371/journal.pone.0267395 (PMC9045837; doi:10.1371/journal.pone.0267395)
Supplement: S1 Text — (DOCX) [file pone.0267395.s009.docx]

**S1 Text. Detailed methodology of recursive covariance selection in graphical modeling.**

We conducted recursive covariance selection for each group by means of backward elimination.

Let $\boldsymbol{Y}^{T}=(Y_{1},\ldots,Y_{m})$ denotes a random vector with $\boldsymbol{y}^{T}$ as its realization. We assume $\boldsymbol{Y}$ is centered and normally distributed with variance-covariance matrix $\boldsymbol{\Sigma}$

$$\boldsymbol{Y}\sim N(\boldsymbol{0},\boldsymbol{\Sigma})$$

, where $\boldsymbol{0}$ is a vector whose components are all 0.

The correlation of $Y_{i}$ and $Y_{j}$ given the other variables except to $Y_{i}$ and $Y_{j}$ is called the partial correlation of $Y_{i}$ and $Y_{j}$, and is defined as

$$Cor\left( Y_{i},Y_{j} | \boldsymbol{Y}_{-\left( i,j \right)} \right)$$

. $\boldsymbol{Y}_{-\left( i,j \right)}$ denotes the other variables except to $Y_{i}$ and $Y_{j}$. The partial correlation is given by the inverse variance-covariance matrix $\boldsymbol{\Sigma}$, let

$$\boldsymbol{K}=\boldsymbol{\Sigma}^{-1}$$

and its elements be $k_{ij}(i=1,..,m;j=1,\ldots,m)$, then

$$Cor\left( Y_{i},Y_{j} | \boldsymbol{Y}_{-\left( i,j \right)} \right)=-\frac{k_{ij}}{\sqrt{k_{ii}}\sqrt{k_{jj}}}$$

holds. The partial correlation matrix is related to graphical modeling. $Cor\left( Y_{i},Y_{j} | \boldsymbol{Y}_{-\left( i,j \right)} \right)=0$ is interpreted as there is no edge between $Y_{i}$ and $Y_{j}$.

By setting the elements of partial correlation matrix to 0 recursively, the model selection can be conducted. Concretely, setting partial correlation of $Y_{i}$ and $Y_{j}$ be 0 means delete an edge between $Y_{i}$ and $Y_{j}$.

Backward elimination is the recursive model selection method, the algorithm is as follows. First, calculate the partial correlation matrix and find the minimum element. Second, eliminating the edge (setting the minimum element to 0). Third, re-estimate the partial correlation matrix with the restrictions. Fourth, repeat these steps. Finally, determine the model when the model criteria GFI is small to some level. After the model selection, we constructed the graphical modeling.[1] The GFI is a measure of the explanatory power of one model, usually ranging from 0 to 1. If closer the GFI is to 1, it has better the explanatory power. Deviance is also a goodness-of-fit statistics for a model, and it is often used for statistical hypothesis testing.

**References:**

1. Epskamp S, Waldorp LJ, Mõttus R, Borsboom D. The Gaussian Graphical Model in Cross-Sectional and Time-Series Data. Multivariate Behavioral Research. 2018;53(4):453-80. doi:10.1080/00273171.2018.1454823.
