# Supplementary material for: Changes in social environment due to the state of emergency and Go To campaign during the COVID-19 pandemic in Japan: An ecological study
Source: PLoS One. 2022 Apr 27;17(4):e0267395. doi: 10.1371/journal.pone.0267395 (PMC9045837; doi:10.1371/journal.pone.0267395)
Supplement: S2 Text — (DOCX) [file pone.0267395.s010.docx]

**S2 Text. The result of influence analysis.**

In period 1 (deviance = 18.34; degrees of freedom = 30; Goodness of Fit Index (GFI) = 0.95), inhabitants in their twenties to fifties was directly correlated with COVID-19 in period 1 (partial correlation = 0.28) (Fig 3A). In period 2 (deviance =32.21; degrees of freedom = 43; GFI = 0.94), the correlation between COVID-19 in period 2 and inhabitants in their twenties to fifties was not significant, whereas, COVID-19 in period 2 was strongly correlated with COVID-19 in period 1 (partial correlation = 0.69) (Fig 3B). In period 3 (deviance =29.07; degrees of freedom = 43; GFI = 0.94), the direct correlation association between COVID-19 in periods 2 and 3 was not significant. (Fig 3C). In period 4 (deviance = 27.83; degrees of freedom = 42; GFI = 0.94), similar to the results of main analysis, COVID-19 in period 4 was directly correlated with COVID-19 in period 3 (partial correlation = 0.41) and indirectly with companies through COVID-19 in period 3(Fig 3D). Similar to the results of the main analysis, the graph for period 5 had the most complicated structure of associations among variables (deviance = 22.62; degrees of freedom = 35; GFI =0.94). In addition, COVID-19 in period 5 was correlated with mobility from rural areas (partial correlation = −0.41, respectively) (Fig 3E).
