# Supplementary material for: Changes in social environment due to the state of emergency and Go To campaign during the COVID-19 pandemic in Japan: An ecological study
Source: PLoS One. 2022 Apr 27;17(4):e0267395. doi: 10.1371/journal.pone.0267395 (PMC9045837; doi:10.1371/journal.pone.0267395)
Supplement: S3 Text — (DOCX) [file pone.0267395.s011.docx]

**S3 Text. The result of sensitivity analysis.**

The structure of models of 0-day lag and 7-day-lag were not completely the same as those of main analysis, however, primary two changes represented in main results were confirmed in both sensitivity analysis as follows. First, in period 2, the number COVID-19 infections in period 1 was correlated with COVID-19 in period 2 (S4 Fig B and S5 Fig B). In period 3, the direct correlation between COVID-19 in periods 2 and 3 was not significant (S4 Fig C and S5 Fig C). Second, in period 5, the correlation of COVID-19 in the present period with that in the previous period was not significant. All variables of environmental factors were correlated with COVID-19 in period 5 and with mobility (S5 Fig E and S5 Fig E).
