# Supplementary material for: Changes in social environment due to the state of emergency and Go To campaign during the COVID-19 pandemic in Japan: An ecological study
Source: PLoS One. 2022 Apr 27;17(4):e0267395. doi: 10.1371/journal.pone.0267395 (PMC9045837; doi:10.1371/journal.pone.0267395)
Supplement: Attachment — Submitted filename: Response to Reviewers_Kanamori et al.docx [file pone.0267395.s012.docx]

DATE: January 18, 2022

Michele Tizzoni

Academic Editor

*PLOS ONE*

PONE-D-21-31460:

Manuscript title: Changes in social environment due to the state of emergency and Go To campaign during the COVID-19 pandemic in Japan: an ecological study

Authors: Kanamori et al.

Dear Tizzoni,

Thank you for the careful review of our manuscript ”Changes in social environment due to the state of emergency and Go To campaign during the COVID-19 pandemic in Japan: an ecological study”, which we submitted to *PLOS ONE*.

We have considered the helpful comments from the reviewer and the editor, and we have revised the manuscript in response to these comments. The changes made are highlighted in the manuscript, and a detailed point-by-point response is described on the following pages of this letter.

Regarding Data Availability, datasets are available on <https://doi.org/10.5061/dryad.f1vhhmgzh>. However, the data sets for the human mobility data sharing will not be publicly available because the human mobility data contains privacy issues and do not allow for publication. This mobility data provided by LocationMind xPop is a proprietary product aggregated from sensitive location information by individual users and only licensed to specific institutions by the data provider. According to the licensing agreement, publication of any visualization of the data requires review by the data provider beforehand in order to ensure data privacy, therefore the redistribution or publication of the data itself is prohibited. For data requests for human mobility data, please contact Mobile Big Data Division at LocationMind company ([data_clearance@locationmind.com](mailto:data_clearance@locationmind.com)).

We hope that, with the revisions described herein, the article is now acceptable for publication in *PLOS ONE*.

Thank you for taking the time to consider the manuscript. I look forward to hearing from you soon.

Sincerely Yours,

Shuko Nojiri

Medical Technology Innovation Center, Juntendo University

2-1-1 Hongo, Bunkyo-ku, Tokyo 113-8421, Japan

Phone No: 81-3-3830-3704

Email Address: [s-nojiri@juntendo.ac.jp](mailto:s-nojiri@juntendo.ac.jp)

**Journal Requirements**

1. Please ensure that your manuscript meets PLOS ONE's style requirements, including those for file naming. The PLOS ONE style templates can be found at

https://journals.plos.org/plosone/s/file?id=wjVg/PLOSOne_formatting_sample_main_body.pdf and

<https://journals.plos.org/plosone/s/file?id=ba62/PLOSOne_formatting_sample_title_authors_affiliations.pdf>.

***Author response:*** We followed the requirements referring templates.

2. In your Data Availability statement, you have not specified where the minimal data set underlying the results described in your manuscript can be found. PLOS defines a study's minimal data set as the underlying data used to reach the conclusions drawn in the manuscript and any additional data required to replicate the reported study findings in their entirety. All PLOS journals require that the minimal data set be made fully available. For more information about our policy, please see <http://journals.plos.org/plosone/s/data-availability>.

Upon re-submitting your revised manuscript, please upload your study’s minimal underlying data set as either Supporting Information files or to a stable, public repository and include the relevant URLs, DOIs, or accession numbers within your revised cover letter. For a list of acceptable repositories, please see http://journals.plos.org/plosone/s/data-availability#loc-recommended-repositories. Any potentially identifying patient information must be fully anonymized.

Important: If there are ethical or legal restrictions to sharing your data publicly, please explain these restrictions in detail. Please see our guidelines for more information on what we consider unacceptable restrictions to publicly sharing data: http://journals.plos.org/plosone/s/data-availability#loc-unacceptable-data-access-restrictions. Note that it is not acceptable for the authors to be the sole named individuals responsible for ensuring data access.

We will update your Data Availability statement to reflect the information you provide in your cover letter.

***Author response:*** We uploaded all minimal datasets on <https://doi.org/10.5061/dryad.f1vhhmgzh> and they will be available when proceeded to publication (until accepted, available on <https://datadryad.org/stash/share/bqdWcDN97dt5HvVN_Up5KgWmAv-T_ZVmG3JUUFpRI2g>).However, the data sets for the human mobility data sharing will not be publicly available because the human mobility data contains privacy issues and do not allow for publication. This mobility data provided by LocationMind xPop is a proprietary product aggregated from sensitive location information by individual users and only licensed to specific institutions by the data provider. According to the licensing agreement, publication of any visualization of the data requires review by the data provider beforehand in order to ensure data privacy, therefore the redistribution or publication of the data itself is prohibited. For data requests for human mobility data, please contact Mobile Big Data Division at LocationMind company ([data_clearance@locationmind.com](mailto:data_clearance@locationmind.com)).

3. PLOS requires an ORCID iD for the corresponding author in Editorial Manager on papers submitted after December 6th, 2016. Please ensure that you have an ORCID iD and that it is validated in Editorial Manager. To do this, go to ‘Update my Information’ (in the upper left-hand corner of the main menu), and click on the Fetch/Validate link next to the ORCID field. This will take you to the ORCID site and allow you to create a new iD or authenticate a pre-existing iD in Editorial Manager. Please see the following video for instructions on linking an ORCID iD to your Editorial Manager account: <https://www.youtube.com/watch?v=_xcclfuvtxQ>.

***Author response:*** The corresponding author has worked on this requirement.

4. We note that Figure S1 in your submission contain [map/satellite] images which may be copyrighted. All PLOS content is published under the Creative Commons Attribution License (CC BY 4.0), which means that the manuscript, images, and Supporting Information files will be freely available online, and any third party is permitted to access, download, copy, distribute, and use these materials in any way, even commercially, with proper attribution. For these reasons, we cannot publish previously copyrighted maps or satellite images created using proprietary data, such as Google software (Google Maps, Street View, and Earth). For more information, see our copyright guidelines: http://journals.plos.org/plosone/s/licenses-and-copyright.

***Author response:*** Thank you for bringing it to our attention. We got permission from copyright holder to publish map under the CC BY 4.0 license using the recommended Permission form.

**Reviewers’ Comments**

**Reviewer #1**

This paper investigates the impact on COVID-19 spreading of the interventions implemented by the Japanese Government to stimulate the internal economy during 2020. Graphical modeling is used to understand the relationship between a set of variables (including mobility, socio-demographic factors, and weather information) and the number of cases reported in different prefectures of Japan. The methodology is applied to 5 different periods of 2020 to study the variation in the relationships between the features studied and the impact on COVID-19 spreading of the government interventions. The natural experiment provided by the implementation of the policies described is undoubtedly interesting and worth studying. Also the approach proposed by the authors is interesting and seems suitable to the purpose. Nonetheless, I think the paper needs substantial changes before being considered for publication. Below, I provide a list of the points that I think should be addressed by the authors. I will cite the line of the paper as L followed by the number of the line I’m referring to.

**Major issues**

1) The Introduction explains very well the interventions implemented by the Japanese government to stimulate the internal economy. However, I think a proper framing within existing literature is missing, apart from a couple of very general sentences. Previous influential studies dealt with similar problems (i.e., understand the role of certain points of interest such as restaurant etc in the spreading), such as:

https://www.nature.com/articles/s41586-020-2923-3

https://www.nature.com/articles/s41562-020-0931-9

On a more general basis, there is a vast literature on the impact of Non-pharmaceutical interventions (especially those impacting mobility) on COVID-19 spread, here a review that can help navigating it:

https://pubmed.ncbi.nlm.nih.gov/33612922/

More work have to be done to place the work in the existing literature

***Author response:*** Thank you for your comments. We revised and added some specific literatures related to our research interest to the Introduction on page 5 (L75-L87).

2) L103, authors state that the incubation period of COVID-19 is 14 days. This is wrong, also the references provided in the paper contradicts this point. Indeed, the average incubation period for COVID-19 (pre-Delta strains) is somewhere between 3 and 5 days. This value is used to define the 5 periods of the analysis, therefore this should be revised according to the right estimate.

Nonetheless, I am not sure if the incubation period is the right quantity to define the ‘lag’ between periods (by ‘lag’ I mean the number of days that are considered as a buffer between periods, and that is now set to 14 days). Indeed, there may also be a delay linked to the reporting of cases. For example, a person infected today will start to present symptoms after the incubation period but may be notified after because of delays associated with the surveillance system. Therefore, besides choosing an appropriate value I think it is essential that authors present as Supplementary Information some sensitivity analysis of the results for different lag values (what happens to the inferred graph if we introduce no lag? Are they stable to this change? What if the lag is equal to the incubation period? What if we also account for delays in reporting? Using relatively small lags should not affect the results much).

***Author response:*** Thank you for your comments. The reason why we selected 14 days as lag (buffer between periods) described in the manuscript is not correct. We considered the sum of time of incubation period, testing and reporting. As you mentioned, we assumed reporting delay of PCR test existed, which was reported around 3 days last year because of immature system of investigation, and people could take PCT tests after presenting symptoms in accordance with the guidance in Japan. Incubation periods is estimated as “on average 5-6 days, however it can be up to 14 days” according to the Situation report (<https://apps.who.int/iris/handle/10665/331686>) from WHO. Considering time above, we applied 14 days.

We corrected and added the reason noted above to the Methods on page 7-8 (L113-L121). In addition, we conducted sensitivity analysis applying 0-day lag and 7-day lag, and added the results to Supporting information. The structure of each graph was not completely the same, but the key results (primary two changes) were consistent.

3) The definition “places where contacts could accrue” is vague even though it is a pretty important point for the paper. How are these places selected? I see that in Table 2 some categories of places are mentioned such as “Business” and “Life”. Why have some categories been chosen and not others? Within a given category, how do you choose some places and not others? The choice of places considered hotspots for COVID-19 transmission should be supported by evidence and references.

***Author response:*** Thank you for your comments. We extracted these places from subcategories of each main category as considered as hotspots for COVID-19 transmission by increased social contacts and mobility in daily life. For example, since there are a lot of subcategories such as attorneys, banks and so on, in main category “Life”, we selected only transportations from it. In addition, they are ordinally closed spaces which have the risk of airborne transmission (restaurants, companies and transportations) and are also related to travel (transportations and tourist spots). We think this selection is logical, because in previous studies, Purwanto et al. reported that hotspots for COVID-19 transmission were places including business facilities, transportations, entertainment, and food venue, and Nishiura et al. reported closed environments were associated with secondary transmission of COVID-19. We added these reports as evidence and references to the Introduction on page 5-6 (L81-L84) and Methods on page 11-12 (L161-L164).

4) The paper lacks a proper materials and methods section. This undermines the overall understanding. First, the datasets used should be better explained:

- The TownPage dataset is used to define places where social contacts could accrue. Is it open access? Is it accurate? Is it complete? How is the dataset built? What are the categories listed and the type of places? Can you provide a plot showing the distribution of different points of interest in the prefectures? Please discuss

***Author response:*** The TownPage dataset is telephone directories of business in Japan and open access, which is almost similar to that of the yellow pages. It includes various categories of business; hospital & clinic, dentist, health & beauty, [fashion & cosmetics](https://www.yellowpages.co.th/en/category/Fashion%20and%20Cosmetics), lifestyle, gourmet & restaurants, housing & residence, shopping, travel & accommodation, leisure & sports, transportation, automotive, education, hobby, banquet & ceremony, public & social services, and pets & pet care, and office & business services. Since the total number of place and facilities in Townpage dataset was quite large, it was considered difficult to show the distribution. Instead of that, we created geographical maps of the sum of number of variables from The TownPage dataset in the 47 prefectures as supporting information S2 Figure.

- The LocationMindxPro is used to measure mobility variation. Is it public? Where can the data be found? What is the size of the population over which mobility is computed? Can you show a plot with (even aggregate) mobility trends in Japan during the period of study? Do you work with raw data? If so, do you apply any preprocessing/inclusion criteria? Please discuss

***Author response:*** This data is not publicly available, but a proprietary product. It is the cell phone’s location data of users of application, “Docomo Map Navi”, which include from approximately 400,000 to 500,000 user IDs. We created the graph of mobility trend in Japan defined as inflow from other prefecture during the period of this study as supporting information S3 Figure. Since those data is processed collectively and statistically in order to conceal the private information by NTT DOCOMO Inc., authors did not work with raw data.

- Also, what do you mean exactly by mobility? Do you assign each user a home location and see whether it is seen in other prefectures during the periods? Or do you define trips? If so, what is the duration of the trip? I think more detail on how you deal with mobility data is needed

***Author response:*** In this study, we defined mobility as inflow including trips from other prefectures to the number of subjects in a prefecture, using mobility data (date, departure prefecture, arrival prefecture, and estimated volume) provided by LocationMind xPop. There are some processing procedures of GPS data to create mobility data conducted by LocationMind xPop; (1) dividing into movement and retention; (2) estimating place of residence and work by clustering of activity pattern; and (3) estimating expansion factor. In the process (1), the minimum duration of retention is fifteen minutes and movement is identified as the remaining. In the process (3), the total number of nighttime population (500m mesh population) identified in the national census is taken as the total number, and it is apportioned to mobile GPS users (number of IDs) who live there. Each ID moves with the obtained expansion factor.

Second, also the methods are not properly described:

- What is “recursive covariance selection for each group by means of backward elimination”? (L193) I understand this is the method used to infer the graphs, but how does it work? I think a few more details would help the reader to understand the methodology

***Author response:*** Thank you for this suggestion. Backward elimination is the recursive model selection method, the algorithm is as follows. First, calculate the partial correlation matrix and find the minimum element. Second, eliminating the edge (setting the minimum element to 0). Third, re-estimate the partial correlation matrix with the restrictions. Fourth, repeat these steps. Finally, determine the model when the model criteria GFI is small to some level. After the model selection, we constructed the graphical modeling. We described more detailed methodology including sentences in the Supporting information (S1 Text). We also created the process of the recursive covariance selection of period 1 in main analysis as S2 Table, which can help readers understand.

- What are the Goodness of Fit Index (GFI) and deviance? (L194) How are they defined and what is their interpretation?

***Author response:*** The GFI is a measure of the explanatory power of one model, usually ranging from 0 to 1. If closer the GFI is to 1, it has better the explanatory power. Deviance is also a goodness-of-fit statistics for a model, and it is often used for statistical hypothesis testing. We added sentences above to the Supporting information (S1 Text).

- L188, is the definition of the group of factors and of outcomes something that influences the graphical modeling or is it just a definition from the authors? Why is Mobility an outcome and not a factor influencing COVID-19 cases?

***Author response:*** The groups of factors and outcomes are different completely from explanatory variables or outcome variable in the regression context. (This is the term of graphical modeling.) We added following sentence to the Methods on page 15 (L205-L208) “In this analysis, considering what variables have edges with the infectiousness number indicates that the infectious number is the outcome variable, and the other variables, including mobility, are explanatory variables in the regression analysis.”

**Minor issues**

1) the very first lines (L45-50) of the paper needs some basic citations (the origin in Wuhan, the declaration of Pandemic by the WHO, the number of REPORTED cases by February 2021)

***Author response:*** Thank you for your comments. We added citations on page 4 (L47-L52).

2) At L52 authors state that SARS-CoV-2 is highly infectious because of its route of transmission. This is not entirely true, it is highly infectious also because of other reasons, such as asymptomatic and pre-symptomatic transmissions

***Author response:*** Thank you for your comments. We corrected the reason of highly infectious as you mentioned with a reference on page 4 (L53-L55).

3) L142, how do you distinguish urban and rural areas?

***Author response:*** As described in Table 2, we define that urban areas are prefectures with ordinance-designated cities which have population greater than 500,000 and have been designated under the local autonomy law, and Tokyo, and that rural areas are other prefectures not categorized as urban areas.

4) L175, I do not understand the need to first compute a seven-days moving average for humidity and temperature and then an average over the all period. Why not directly average over the period?

***Author response:*** Since it was so complicated to extract 1-day data from the data source, we extracted data by one-week unit, computed a 7-day moving average and averaged them over the period. We think this way is almost the same as the direct average.

5) L177, why is mobility not expressed per 100,000 as other variables?

***Author response:*** Since we aim to investigate the impact of inter-prefecture mobility only including inflow, we think it is reasonable to use total volume of human mobility directly.

6) L165, why add the number of inflow people to the people in the prefecture?

***Author response:*** Because we think that using inflow mobility from other prefectures could reflect the impact of government policies better than intra-prefecture mobility which we think is to reflect mobility occurring in daily life. For example, during Go To campaign, it is ordinally considered that more people try to travel a bit far away from their living places.

7) I find the results very difficult to read, now the section is just a long list of sentences like this one “inhabitants in their twenties to fifties showed a partial correlation with COVID-19, having a partial correlation with tourist spots, which was correlated with restaurants and variables of mobility?” Besides just commenting on the graphs I think the authors should also make an effort to provide some interpretation behind this or use additional plots to help the reader follow the logic.

***Author response:*** We revised Figure 2 & 3 and their description in the Results using symbols to follow them more efficiently.

8 ) L334, “we did not take the intercorrelation among the samples (prefectures) into account when constructing the graphical model” How can this affect the results?

***Author response:*** This analysis ignored the intercorrelation of the samples. For example, the infectious number of one prefecture may influence the adjacent prefectures directedly, but we assume all influence is thorough other variables such as the mobility or the pre-period infectious number.

If there is the intercorrelation of the samples, it varies the graphical structures. However, it is not great impact because the influence from other prefectures have lags and seems to be almost explained by mobility.

9) Among the limitations, also the possible biases coming from the datasets (especially mobility ones) used should be discussed

***Author response:*** Thank you for your comments. The possible biases from the datasets exist in the following points. First, we applied TownPage datasets extracted in August 2020, however, the number of facilities might be slightly changed through the study period due to closing or newly opening. Second, the mobility data is estimated volume calculated from GPS data. We added these biases to the Limitations on page 24 (L370-L373).

**Reviewer #2:** The study investigates the effects of the state of emergency and go-to travel policies on the number of COVID-19 cases in Japan. This study concludes that the state of emergency affected the control of COVID-19 spread and that the go-to campaign increased the number of cases by changing the locations of social contacts. The objective of the study is well motivated -- to provide evidence for lockdown policies based on big mobility data, which could be useful for policy makers. However, the statistical analysis (which the methods are very unclear) are very weak and lacks the rigor to reach any conclusions on the impact of the policies on the number of infections.

Here are some comments that I recommend the authors to address:

- The methodological procedure of the analysis is very hard to follow. I strongly recommend the authors to provide more detail on the statistical analysis method they used to produce the network plots. Are we looking at just correlations between variables? or are we able to capture any causal structures between them?

***Author response:*** Thank you for your comments. We’re looking at just correlation between variables. We described more detailed statistical analysis method in the Supporting information (S1 Text).

- page 17 line 241 what does this mean? "The directed edge (-) from the variables in the group of factors to the variables in the group of outcomes indicates the time order." How can we tell the 'time order'? and what do you mean by 'time order'?

***Author response:*** We assumed that there was “Time order” between variables in the group of factors and those in the group of outcomes, which means factors are antecedent to outcomes in time. Thus, causal inference can be determined. In the process of graphical modeling, categorizing variables into these two groups is necessary and taken into account to the model selection.

- Figure 2 & 3 are visually very hard to read. Please update using more high resolution figures.

- Overall, the analysis is very weak (merely captures time-lagged partial correlation only?) and thus the discussions/conclusions are not convincing. At least some econometric analysis that can give statistical conclusions on the effects of covariates on the dependent variable is needed; there are already many studies that use epidemiological models (e.g., SIR, SEIR) to rigorously achieve these kinds of analysis, which may be a path that the authors could pursue.

***Author response:*** We revised Figure 2& 3 and their description in the Results using symbols to follow them more efficiently. We also revised the Discussions and Conclusions to be more convincing.

Thank you for your suggestion on statistical models, but the reason we applied graphical modeling is to investigate the impact of government policies by clarifying the correlations among COVID-19 cases, mobility and environmental factors in five periods. This statistical method is useful to explain correlations among variables clearly by visualizing the conditional independence structure in a joint distribution. By contrast, mathematical, epidemiological models such as SIR are simulation models of dynamics of infectious disease taking into account population and time, and they cannot include covariates in the analysis and reflect the public health intervention.
